# Revisiting the Health Insurance Portability and Accountability Act: Practical Applications for the Modern Plastic Surgeon

**DOI:** 10.1093/asjof/ojag096

**Published:** 2026-05-30

**Authors:** Myiah P Quach, Mary Kristine Carbullido, Jasmine N Craig, Peter J Wirth, Ellen C Via, Venkat K Rao

## Abstract

The protection of sensitive patient information is central to modern healthcare delivery and is legally established through the Health Insurance Portability and Accountability Act (HIPAA). For plastic surgery practices, HIPAA compliance presents unique and increasingly complex challenges regarding clinical photography, public-facing marketing, and digital communication. We conducted a focused review to synthesize essential regulatory principles, common pitfalls, and practical strategies for maintaining compliance when creating or operating a plastic surgery practice. We outline historical developments of HIPAA, foundational Privacy and Security Rule requirements, and considerations specific to photography, metadata, electronic communication, and patient consent. Further, we describe common violations encountered in routine workflows and the associated civil and criminal penalties enforced by the Office for Civil Rights. By providing practical, specialty-specific guidance, this article aims to help plastic surgeons strengthen patient privacy protections without compromising clinical efficiency, thus reinforcing the trust at the core of the patient–provider relationship.

**Level of Evidence:** 5 (Risk) 
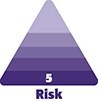

The protection of sensitive patient information is the foundation upon which our modern healthcare system is built. This protection, legally encompassed by the Health Insurance Portability and Accountability Act (HIPAA), allows patients the opportunity to be treated and billed transparently.^1^ Further, the legal precedence set by HIPAA serves to standardize how healthcare systems facilitate healthcare in the age of rapidly evolving technology. Adherence to and understanding of HIPAA are critical as failure to comply results in breaches of patient privacy, mistrust by the public, and severe financial and legal penalties.

Although HIPAA applies to all healthcare providers, its application in the context of plastic surgery presents a unique challenge because of the specialty's heavy reliance on photography with digital and public-facing marketing. Here, we present guidance that is both easy-to-follow and specifically tailored to the workflow of a plastic surgery practice. This paper aims to develop practical compliance strategies to promote increased patient privacy protection without compromising clinic efficiency.

This article is meant to provide an overview of general principles associated with current HIPAA compliance, including common pitfalls and their consequences. This guidance is not intended to provide comprehensive legal advice and should not be used as a substitute for formal legal consultation.

## METHODS

We conducted a focused narrative review to synthesize key principles of HIPAA compliance relevant to plastic surgery practice creation. A structured search of publicly available sources was conducted in September and October 2025 using PubMed, Google Scholar, and the US Department of Health and Human Services (HHS) website. Search terms included “HIPAA Privacy Rule,” “HIPAA Security Rule,” “Breach Notification Rule,” “HITECH Act,” “Omnibus Final Rule,” “clinical photography,” “medical photography privacy,” “plastic surgery social media HIPAA,” and “HIPAA penalties.” Additional references were identified by manual review of references from key articles.

Because the goal of this review was to summarize regulatory guidance and practical considerations rather than perform a comprehensive systematic review, the search strategy was designed to identify representative regulatory documents, policy statements, and peer-reviewed literature relevant to clinical practice. Sources were included if they contained primary regulatory language, official guidance on HIPAA implementation, documented enforcement actions, or practical recommendations applicable to plastic surgery workflows. Data collected for this article was further reviewed by clinicians with experience in plastic surgery practice management and privacy compliance.

## RESULTS

The search strategy identified regulatory documents, federal guidance materials, and peer-reviewed publications relevant to HIPAA compliance in clinical practice. Priority was given to primary regulatory documents, Office for Civil Rights (OCR) guidance and enforcement materials, and peer-reviewed publications addressing privacy considerations in surgical workflows, clinical photography, electronic communication, and social media use.

Included sources (*n* = 23) spanned the original HIPAA statute and subsequent regulatory developments, including the Privacy Rule, Security Rule, Breach Notification Rule, Health Information Technology for Economic and Clinical Health (HITECH) Act provisions, and the Omnibus Final Rule. Government guidance documents were used to clarify risk analysis requirements, business associate (BA) agreements (BAAs), and breach reporting obligations. Peer-reviewed surgical literature contributed to discussion of clinical photography, metadata management, electronic communication, and social media use within plastic surgery.

Synthesis of these sources revealed 4 primary thematic domains relevant to plastic surgery practice: (1) the historical and regulatory evolution of HIPAA enforcement; (2) administrative and technical safeguards required under the Security Rule; (3) risks specific to clinical photography and embedded metadata; and (4) compliance considerations specific to plastic surgery related to marketing, social media, and electronic communication.

## DISCUSSION

### Historical Overview of HIPAA

The HIPAA, passed in 1996, was originally enacted with 2 goals: (1) to protect employees from loss of health insurance when transitioning between jobs and (2) to prevent healthcare fraud by standardizing the use of electronic protected health information (PHI).^2,3^ Following initial implementation, the HHS passed the Privacy Rule effective in April of 2003.^3^ The HIPAA Privacy Rule defined PHI as any individually identifiable health information relating to a person's physical or mental health, provision of healthcare, and payment for that care and thus may include identifiers such as names, dates, phone numbers, photographs, medical records, and diagnoses, among others.^3,4^ The Privacy Rule, further, went on to stipulate when authorization is required for use by the healthcare provider and gave individuals the right to access their own records.^3^

In April 2005, the Security Rule was enacted to create specific actionable safeguards related to the protection of electronic PHI. This included safeguards related to password management, device security, data backups, secure disposal, automatic log-off, and contingency planning. Enforcement of compliance was possible throughout HIPAA's history; however, standardization for investigation was created with the Breach Enforcement Rule, effective in 2006. In addition, this rule defined how the HHS would issue civil monetary penalties for noncompliance. The Breach Notification rule, effective in 2009, expanded on the Enforcement Rule by requiring all breaches of PHI be reported to affected individuals and the Department of Health and Human Services’ (DHHS) OCR.^5^

In 2009, the HITECH Act was created to promote the use of health information technology and enforce significant monetary penalties for HIPAA violations.^6^ The final major amendment to the HIPAA legislation came into effect in 2013, called the Omnibus HIPAA Final Rule.^7^ This rule bolstered many existing protections and clarified the use of PHI in marketing, research, and fundraising by increasing the consent requirements for photographs and videos.^7^ The Omnibus Final Rule further made individuals directly liable for HIPAA compliance and forced disclosure of “presumed” breaches, no longer limited to confirmed breaches of confidentiality.^8^

### General HIPAA Considerations

Any individual or organization that directly engages with PHI is considered a covered entity under HIPAA.^9^ The first step for any HIPAA-covered entity in its effort to comply with the standards set by HIPAA's Security Rule includes risk analysis that provides the organization with detailed information regarding risks to the “confidentiality, integrity, and availability of PHI held by the organization.”^10^ This is accomplished by identifying how PHI is stored, maintained, or transmitted and any potential threats to it. Protecting this information is accomplished by encrypting the data and keeping any hard drives or external data devices physically secure. Risk analyses must be conducted before the opening of a practice and regularly reviewed and updated as the practice grows. The Security Risk Assessment Tool at HealthIT.gov assists practices when conducting risk assessment and provides further guidance on this process.^11^

For plastic surgery practices, this initial risk analysis often reveals a higher-than-average volume of image-based PHI, including standardized preoperative and postoperative photography. Because images are central to documentation, outcomes tracking, and aesthetic assessment, plastic surgery workflows frequently involve storage, transmission, and potential external sharing of visual data, increasing the complexity of safeguarding ePHI compared with many non–procedure-based specialties. In practice, this means that surgeons opening a new plastic surgery practice must proactively map how photographs, consultation documentation, billing information, and digital communications are integrated in their workflow. Early identification of potential points of vulnerability allows safeguards to be built proactively rather than retrofitted following a breach.

After initial risk assessment, ensure that all technology used by the covered entity is HIPAA compliant. This includes choosing a HIPAA compliant, password-protected electronic medical record (EMR) keeping system, telemedicine system, and patient portal system. These services are often supported by independent contractor third-party vendors also known as BAs. Each BA is required to provide the covered entity a BAA that frames the use of PHI by the BA, including describing the permitted and required uses and disclosures of PHI, required security measures in order to protect PHI, and steps to take to destroy or return PHI in the event of contract termination.^12^ Additional steps to consider during the protection of electronic PHI may include encryption of Wi-Fi, increasing password complexity requirements for PHI access, and automatic computer log-off.

It is important to designate 1 staff member as a security officer who will be responsible for creating written policies and procedures ensuring adherence to the standards set by HIPAA.^13^ This person should also undertake the responsibility of training the rest of the staff on these HIPAA-compliant policies, which include but are not limited to how PHI is maintained at workstations, legal access of patient files, authorization of PHI release, and proper disposal of PHI. This also includes training employees on what constitutes a breach and that all suspected breaches should be reported to the appropriate management team immediately to comply with the Breach Notification Rule.^14^ HIPAA training should be repeated annually for all staff to ensure privacy and security. However, even the best laid plans often cannot cover every scenario, and a practice manager may consider obtaining insurance for the significant financial penalties following a breach.

### HIPAA Considerations Specific to Plastic Surgery

The increasing integration of digital communication and social media into plastic surgery practice has amplified the importance of clear privacy protections. Previous literature in plastic surgery has highlighted both the educational and marketing benefits of online engagement, while simultaneously raising medicolegal concerns regarding patient privacy, consent, and professional boundaries.^15-17^ Within this evolving digital environment, HIPAA compliance represents a foundational framework that helps define the boundaries between appropriate educational or marketing use of clinical information and the protection of patient privacy.

#### Patient Consent

**Figure 1.**
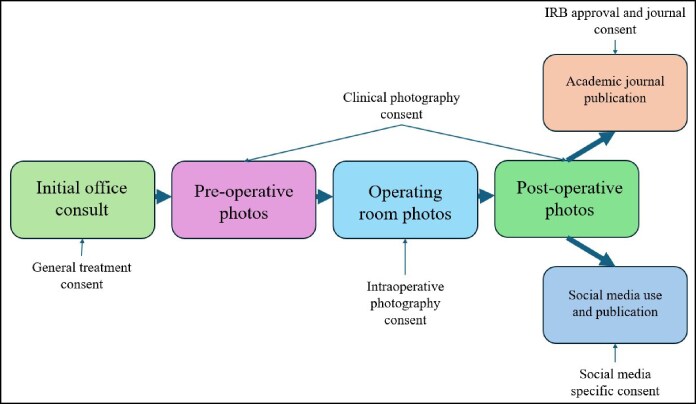
Flowchart indicating the steps in which consents are required.

General surgical and procedural consents often include a clause regarding the use of photography or recordings for quality improvement and education (Figure 1). In plastic surgery, however, photography often extends beyond internal documentation to public-facing marketing, portfolio development, and social media engagement. Because aesthetic outcomes frequently influence patient decision making, surgeons must be particularly deliberate in distinguishing between consent for clinical documentation and separate authorization for public-facing use. This may apply to PHI utilization between covered entities such as healthcare providers, insurance plans, clearinghouses, and other healthcare BAs. These release authorization forms should specify what kind of PHI will be disclosed, who may make and receive the disclosure, and an expiration date.

When releasing PHI to noncovered entities, such as social media platforms or activities outside of immediate healthcare business, the HIPAA Privacy Rule requires a separate written authorization.^15^ It is thus critical to create a separate consent form clearly designating the use of PHI for social media use.^16^ Social media release forms may include a listing of the social media platforms and information relating to the diagnosis or treatment. Additionally, the release should specify to patients that unlike a general consent form which usually includes the right of the patient to revoke consent at any time, this is impossible to guarantee given the nature of social media.^16,17^ Patients need to be made aware of the risk that their content may be made permanent through reposts or manipulation from outside parties. Patients should be counseled that signing the release is voluntary, there will be no compensation, and they always retain the right to revoke the release. This form may also address the use of the patient's “likeness” in imaging, as this is considered their property. The permanence of posts on the internet and further manipulation outside of the control of the initial disclosing party should be mentioned.

#### Photography

Unlike many other surgical specialties, plastic surgery relies heavily on standardized preoperative and postoperative photography for documentation, outcome assessment, and marketing. This increases the likelihood that images will be shared outside the traditional medical record, amplifying the risk of inadvertent PHI disclosure. Although a patient may authorize the use of their PHI in social media, it remains best practice to maintain their privacy, when possible, by de-identifying social media posts as much as possible. Consistently de-identifying shared content helps to maintain patient dignity and foster trust between the practice and both current and prospective patients.

Identifiers described by the HIPAA Privacy Rule include names, medical record numbers, addresses, telephone numbers, email addresses, web addresses, social security numbers, health plan numbers, and account numbers.^18^ Any type of date (such as date of birth, service, procedure, admission, or discharge) is also an identifier.^19^ This is an important consideration for surgeons who may wish to post content on the day of surgery.

Although there are no photographic identifiers defined by HIPAA or the Privacy Rule that must be removed to protect a patient's privacy, reasonable effort must be made to remove features or characteristics that may allow identification of an individual. This may include anatomic anomalies, birthmarks, scars, unique clothing, jewelry, or tattoos.^19^ Specifically, for full-faced photographs, “eye bars” are often insufficient for deidentification, as many people may still be recognizable. When use of full-face photographs is necessary, ensure the patient has been fully informed that they may still be recognized even after all reasonable privacy measures have been taken.

Aesthetic practices may have increased reliance on personal mobile devices, especially if they are outside of a traditional hospital setting. This increases the importance of strict adherence to encrypted applications and secure storage protocols. If photographs are taken on a personal cell phone, use a HIPAA-compliant application associated with the EMR service to ensure secure storage within the encrypted app rather than the smartphone itself. This is critical because smartphones are often linked to cloud-based storage, which is likely not HIPAA-compliant storage and poses a significant risk to patient privacy.^20^ Recommendations on clinical photograph storage by Chandawarkar and Nadkarni include end-to-end encryption during media transfer, verification of all users, 2-factor authentication for storage access, a remote wipe function of media for possible theft, automatic deletion of messages, quick erasure of photographs stored on smartphones after transfer to a secured storage site, and an audit trail.^20^ When using a digital camera, metadata may be removed by clearing geotags and using a password-protected SD card for storage and no internet capability. Further, photographs should be free of all patient identifiers in the file name to ensure security.^20^

All photograph and video files have embedded metadata. Metadata refers to information describing the structural characteristics of the file, such as date, time, and location, among others. This metadata often includes potentially identifiable information and accompanies the file being uploaded to social media, signifying a breach of HIPAA. Specific metadata components that may compromise PHI are the author and file name, editing history, geographic location, and creation date. Although this metadata is not obviously visually attached to the file, it can be accessed when the file is downloaded. Incorporating routine metadata removal into the workflow is thereby a necessary component of responsible digital practice management.

This removal of meta data is performed by going into a file's “properties” and then the “details” tab, which presents options to remove specific embedded properties and personal information. Removing metadata will help ensure the privacy of your patients’ information that may be distributed with the posted images, so they are not accessible by third parties. Additionally, metadata may be removed from photographs when the photograph is converted to a PDF file that is unrecoverable by third parties.

#### Electronic Communication

Electronic communication with patients in the context of plastic surgery care has become an important part of practice in the modern age but should be approached with cognizance of current medicolegal standards.^21^ Plastic surgery practices often receive unsolicited direct messages, preoperative photographs, and requests for cosmetic advice through social media platforms. These interactions may blur the boundary between marketing engagement and clinical consultation, raising questions about when PHI is created and when a provider–patient relationship may be implied. Establishing clear policies for redirecting such communications to secure portals is therefore particularly important in this specialty.

In accordance with HIPAA, provider teams should limit all direct communication with patients to HIPAA-compliant mediums such as a patient portal or encrypted, secure email to avoid nonsecure disclosure of private information. Providers should limit giving specific medical recommendations, especially over nonsecure social networking sites to prevent legal liability.^21^ Patients reaching out to the practice seeking medical advice through nonsecure email, social media sites, or other non-HIPAA compliant means should be directed to set up an account with the practice's patient portal or instructed to set an appointment.

Another form of patient electronic communication that has specific consequences for plastic surgery includes patient reviews and comments published publicly on the internet. Although these negative reviews and comments may be detrimental to a practice, providers are prohibited from responding publicly with any information that identifies the reviewer as a patient of the practice.^22^ Because aesthetic practices are especially sensitive to online reputation and patient perception, the inability to publicly clarify clinical details may be particularly challenging for plastic surgeons whose practices rely heavily on consumer-driven decision making.

### Common HIPAA Violations and Associated Penalties

Common violations of HIPAA compliance often arise from lapses in routine clinical workflows, especially in environments where patient information is frequently accessed or shared. Our recommended guidelines to avoid these common violations are shown in Table 1. One of the most frequent violations is the unauthorized disclosure of PHI by not confirming the patient's identity (eg, full name and date of birth) before releasing information. PHI may be unintentionally disclosed during casual conversation in public areas where persons uninvolved with treatment can overhear. Employees may violate HIPAA by sharing login credentials or by leaving workstations unlocked with PHI visible. Importantly, employees must never enter a patient's chart without a treatment-related purpose (ie, curiosity access). Physical security of PHI is at risk when printed materials such as charts and lists of patient information are left unattended or misplaced, further contributing to compliance failures.

**Table 1.** Guidelines for Plastic Surgery Practices to Optimize HIPAA Adherence

Guidelines
1. Perform the HIPAA-required risk analysis
2. Identify a security officer and train all employees on HIPAA requirements
3. Develop written policies and procedures to protect all PHI
4. Ensure you have business associate agreements from all business associates
5. Develop reporting steps for cases of breach of confidentiality; contact your risk manager or medical malpractice insurance carrier
6. Revisit these steps on an annual basis to ensure full compliance

HIPAA, Health Insurance Portability and Accountability Act.

HIPAA violations are investigated by the OCR of the DHHS. OCR identifies these violations through the conduction of regular compliance reviews and investigation of complaints. Penalties for violations are assessed based on severity and negligence involved.^23^ Minor, unintentional violations within an institution may result in counseling, mandatory retraining, or written warnings. More serious violations, especially those involving repeated improper access, sharing PHI without authorization, or reckless handling of ePHI, may result in civil or criminal penalties. Civil money penalties range from $100 to $50,000 per violation (Table 2).^24^ Criminal penalties are managed by the Department of Justice and apply for knowingly obtaining or disclosing PHI, with fines up to $250,000 and potential imprisonment in cases of malicious intent, commercial gain, or personal profit (Table 3).^24^ Because HIPAA enforcement holds both individuals and institutions accountable, maintaining compliance requires consistent vigilance, education, and adherence to privacy safeguards.

**Table 2.** Varying HIPAA Violation Severity with Associated Civil Penalties.^24^

HIPAA violation	Penalty range per violation	Annual maximum for repeat violations
Unknowing	$100-50,000	$25,000
Reasonable cause	$1000-$50,000	$100,000
Willful neglect but violation is corrected within the required time period	$10,000-$50,000	$250,000
Willful neglect and is not corrected within the time period	$50,000	$1.5 million

HIPAA, Health Insurance Portability and Accountability Act.

**Table 3.** Varying HIPAA Violation Severity with Associated Criminal Penalties.^24^

HIPAA violation	Money penalty maximum	Maximum imprisonment sentence
Offenses committed knowingly to obtain or disclose individually identifiable health information	$50,000	1 year
Offenses committed under false pretenses	$100,000	5 years
Offenses committed with the intent to sell, transfer, or use individually identifiable health information for commercial advantage, personal gain, or malicious harm	$250,000	10 years

HIPAA, Health Insurance Portability and Accountability Act.

### Limitations

This review has several limitations. First, this study represents a narrative review rather than a formal systematic review and therefore may be subject to selection bias in the identification and interpretation of sources. Although a structured search strategy was used to identify relevant literature, it is possible that some relevant sources were not captured. Second, much of the available literature on HIPAA implementation consists of regulatory documents, government guidance, and commentary rather than empirical research studies, limiting the ability to perform quantitative synthesis. Finally, the recommendations presented are intended to help provide practical guidance for clinicians and should not be interpreted as formal legal advice.

## CONCLUSIONS

The practice of plastic surgery is built on the trust fostered between the provider and the patient. Compliance with HIPAA yields more than simple abatement of punitive damages; it allows plastic surgery practitioners to minimize risk while enhancing patient trust. Therefore, it is critical for privacy compliance to be embedded into daily workflow. These guidelines are essential when building a new practice in the ever-evolving digital healthcare landscape.

## Data Availability

Data sharing is not applicable to this article as no new data were created or analyzed in this study.
